# Determining the Factors Predicting the Success of Medical Management in Patients with Ectopic Pregnancy: Medical management in patients with ectopic pregnancy

**DOI:** 10.31661/gmj.vi.3761

**Published:** 2025-06-29

**Authors:** Ayda Roostaee, Zeinab Safarpour Lima, Sima Sharif Kazemi, Ghazaleh Dezyani, Seyed Taleb Pourseyedian

**Affiliations:** ^1^ Department of Radiology, School of Medicine, Iran University of Medical Sciences, Tehran, Iran

**Keywords:** Ectopic Pregnancy, Methotrexate, Human Chorionic Gonadotropin Hormone

## Abstract

**Background:**

Ectopic pregnancy is considered the most common cause of pregnancy-related deaths in the first trimester. Methotrexate is recognized as an effective drug for the treatment of ectopic pregnancy. The aim of this study was to determine the associated and predictive factors for success in medical treatment among patients with ectopic pregnancy (EP).

**Materials and Methods:**

After collection of Demographic information, serum β-hCG levels and ultrasound findings were evaluated and compared between two groups: those with successful medical treatment and those without.

**Results:**

The mean β-hCG level have not significant difference between the two groups (P=0.806). The frequency of previous IUD use in the successful treatment group was 8%, while there were no cases in the failure group; however, this difference was not statistically significant (P=0.547). The frequency of prior EP, the observed frequency of hematoma and the frequency of tubal ring observation, which also showed no significant difference (respectively P=0.9, P=0.9 and P=0.111). Logistic regression analysis revealed that none of the investigated variables were significant predictors of treatment success. However, the presence of a tubal ring (OR: 6.500, 95% CI: 0.799–52.897, p = 0.080) and increased endometrial thickness (OR: 1.317, 95% CI: 0.971–1.786, p = 0.077) showed borderline significance. Commonly considered factors, such as gestational age, β-hCG levels, patient age, parity, and gravidity, did not significantly influence treatment success. The study highlights a high success rate for single-dose methotrexate therapy and the potential utility of tubal ring and endometrial thickness as clinical indicators, warranting further investigation.

**Conclusion:**

The results of this study indicate that single-dose methotrexate treatment for tubal ectopic pregnancy leads to a high success rate. Given the sample size of this study, none of the variables had a significant impact on treatment success or predictive power.

## Introduction

Ectopic Pregnancy (EP) refers to a condition where a developing embryo (blastocyst) implants and grows in tissue other than the endometrial lining of the uterus. Ectopic pregnancies most frequently occur within the fallopian tube. However, they can also develop in other locations, such as the interstitial area, cornua of the uterus, ovaries, cervix, surgical scars, abdominal cavity, or in combination with an intrauterine pregnancy, known as a heterotopic pregnancy [1]. Ruptured ectopic pregnancy is the leading cause of maternal mortality in the first trimester, accounting for 9% to 14% of maternal deaths and representing 5% to 10% of all pregnancy-related deaths [2]. Women with EP may exhibit non-specific symptoms such as lower abdominal pain and vaginal bleeding, which can clinically resemble appendicitis, urinary stones, early miscarriage, or trauma [3].

The current standard for diagnosis includes ultrasound imaging (US)—either transvaginal (TVUS) or transabdominal (TAUS)—and monitoring of beta-human chorionic gonadotropin (β-hCG) levels [4]. Early and accurate diagnosis of EP can help reduce maternal mortality rates. Notably, no identifiable risk factor exists in half of the diagnosed EP cases. Risk factors include a history of EP, fallopian tube damage, prior pelvic surgery, pelvic infections, prior fallopian tube pathology or surgery, infertility, smoking, advanced maternal age (over 35 years), pelvic inflammatory disease, endometriosis, anatomical abnormalities of the reproductive system, pregnancy with an intrauterine device (IUD) in place [5][6]. EPs are particularly challenging and have become more common due to the rise in assisted reproductive technologies (ART). The incidence is approximately 1 in 100 pregnancies involving in vitro fertilization (IVF) and 1 in 7000 pregnancies using ART with ovulation induction [7]. The growing use of IVF has contributed to higher rates of EPs, with studies reporting 2.1%-8.6% of IVF pregnancies experiencing EP after embryo transfer, compared to 2% in spontaneous conceptions [8]. Additionally, the World Health Organization (WHO) highlights a global increase in cesarean sections, now accounting for 21% of all deliveries. This trend is linked to a rising incidence of cesarean scar pregnancies (CSPs), a specific type of EP [9].

After confirming the diagnosis of EP, treatment can be either conservative or invasive, depending on the location of the EP, gestational age, and size of the gestational sac (GS). There are three primary approaches to managing EP: medical treatment, surgical intervention, and expectant management [10]. The current standard for medical management of EP involves intramuscular injection of methotrexate (MTX), a folate antagonist that inhibits rapid cell division, thereby terminating the EP [11]. Additionally, given the significant health risks associated with ectopic pregnancies, timely and effective management is crucial. Methotrexate has emerged as a key medical treatment for unruptured ectopic pregnancies, offering a non-invasive alternative to surgical intervention. However, the efficacy and safety of methotrexate, as well as the factors influencing treatment outcomes, remain areas of active research. Scientific reports estimate the success rate of MTX treatment without the need for surgical intervention at 70% to 95%, although its efficacy is lower in patients with higher initial β-hCG levels. However, recent meta-analyses have shown inconsistent findings regarding the success rates and risk of side effects with different treatment regimens, highlighting the need for further research in this area. Several studies have identified predictive factors that may influence the success of methotrexate treatment in ectopic pregnancies. For instance, a study by Stovall et al. (4) found that lower serum human chorionic gonadotropin (hCG) levels at the time of treatment are associated with higher success rates. Specifically, patients with initial hCG levels below 5,000 mIU/mL had a significantly greater likelihood of successful treatment without the need for surgical intervention[12]. A better understanding of the factors influencing MTX efficacy in EP treatment could help clinicians select the most appropriate therapeutic approaches. Identifying predictors of treatment success could prevent side effects from ineffective therapies, reduce treatment costs, and improve both the physical and mental well-being of patients. Therefore, the purpose of this study was to determine the factors associated with and predictive of successful medical management of EP using MTX.

## Materials and Methods

The study was a case-control design and conducted with the approval of the Medical Ethics Committee of Iran University of Medical Sciences (Ethics Code: IR.IUMS.FMD.REC.1403.159). Patients diagnosed with tubal ectopic pregnancy who had undergone single-dose methotrexate medical treatment at Akbarabadi Hospital in Tehran between 2017 and 2023 were included in the study. These patients were categorized into two groups: those with successful medical treatment (case group) and those with failed medical treatment (control group).

### Definitions of Treatment Outcomes

Patients were categorized into two groups based on treatment outcomes:

• Successful Treatment (Case Group): Defined as a decrease in serum beta-hCG levels to less than 15 mIU/mL within 4-6 weeks following methotrexate administration, without the need for surgical intervention. Successful treatment was also confirmed by follow-up ultrasound showing resolution of the ectopic mass.

• Unsuccessful Treatment (Control Group): Defined as either a failure to achieve the aforementioned decrease in serum beta-hCG levels or the need for surgical intervention (laparoscopy or laparotomy) due to persistent or increasing hCG levels or clinical symptoms indicating complications.

•

### Sample Size and Power Analysis

The study included a total of 100 patients diagnosed with tubal ectopic pregnancy who underwent single-dose methotrexate (MTX) treatment. To ensure that this sample size was adequate for the analyses performed, a power analysis was conducted prior to the study. Based on previous literature, we anticipated an expected success rate of 80% for single-dose MTX treatment. Our goal was to detect a minimum difference of 15% in success rates between the successful and unsuccessful treatment groups. Assuming a significance level (alpha) of 0.05 and a desired power (1 - beta) of 0.80, we calculated the required sample size using the following formula for comparing two proportions:

n=(p1 −p2 )2(Zα/2 +Zβ )2.(p1 (1−p1 )+p2 (1−p2 ))

Where:

• Zα/2 is the Z-value for a two-tailed test at the 0.05 significance level (approximately 1.96).

• Zβ is the Z-value for the desired power (approximately 0.84 for 80% power).

• p1 is the expected success rate in the case group (0.80).

• p2 is the expected success rate in the control group (0.65, assuming a 15% lower success rate).

The power analysis indicated that a minimum sample size of approximately 60 patients per group would be required to achieve adequate power for detecting a significant difference in treatment success rates. However, we acknowledge that our final sample included 60 patients in the case group and only 40 patients in the control group, resulting in a case-to-control ratio that is not optimal. This limitation may affect the statistical power of our comparisons and the generalizability of our findings. Demographic data, including age, gestational age, gravidity, parity, history of ectopic pregnancy in previous pregnancies, use of assisted reproductive technology (ART), and contraceptive methods (e.g., IUD placement), were extracted from archived patient records and documented in the study checklist. Additionally, serum beta-hCG levels and ultrasound findings—such as the size and location of the ectopic pregnancy, presence of hematoma, fluid in the cul-de-sac, tubal ring shape, endometrial thickness, and Doppler findings—were recorded and compared between the two groups. Data analysis was performed using IBM SPSS Statistics for Windows, version 30 (IBM Corp., Armonk, N.Y., USA). Qualitative variables were expressed as frequency and percentage, while quantitative variables were presented as mean and standard deviation. Comparisons between the two groups were conducted using T-tests and Chi-square tests (Fisher’s exact test), with a P-value of less than 0.05 considered statistically significant. Logistic regression was performed to evaluate the association of multiple clinical variables with the likelihood of successful treatment. Univariate analyses were conducted to identify variables significantly associated with treatment success, with those having a P-value of less than 0.1 considered for inclusion in the multivariate logistic regression model. This approach helps ensure that the final model accounts for potential confounders while maintaining statistical power. The final logistic regression model was assessed for goodness-of-fit using the Hosmer-Lemeshow test, and multicollinearity among predictor variables was evaluated using variance inflation factors (VIF), with a VIF value greater than 10 indicating multicollinearity.

## Results

**Table:** Table1. Studied variables in two groups, successful and failed treatment of EP with MTX.

**Variable**		**Successful Treatment Group (**n= 60 **)**	**Failed Treatment Group (**n= 4 **)**	**P-value**
**Patient Age (years, Mean ± SD) **		31.84 ± 4.589	34.50 ± 1.707	0.272
**Parity (Mean ± SD) **		1.54 ± 1.450	2.25 ± 1.50	0.203
**Gravidity (Mean ± SD) **		3.25 ± 1.5	2.5 ± 1.291	0.259
**Gestational Age (days, Mean ± SD) **		37.00 ± 6.633	39.26 ± 12.80	0.732
**History of IUD use (Yes, %) **		5 (8%)	0	0.547
**History of EP (Yes, %) **		9 (15%)	0	0.900
**EP Location **	**Isthmic (Frequency & %) **	4 (6.6%)	4 (6.6%)	0.263
	**Ampullary (Frequency & %) **	56 (93.4%)	3 (75%)	
**EP Size (Mean Diameter, Mean ± SD) **		15.41 ± 5.01	18.63 ± 6.15	0.237
**Tubal Ring (Observed, %) **		52 (86%)	2 (50%)	0.111
**Hematoma Presence (Observed, %) **		32 (53%)	2 (50%)	0.900
**Cul-de-sac Fluid (Observed, %) **		34 (56%)	3 (75%)	0.632
**β-hCG Level (Mean ± SD) **		1395 ± 1464	1135 ± 481.5	0.806
**Endometrial Thickness (mm, Mean ± SD) **		11.17 ± 5.35	6.50 ± 2.64	0.090
**Uterine Artery Pulsatility Index (PI) **	**Left (Mean ± SD) **	2.119 ± 0.883	2.119 ± 0.883	0.271
	**Right (Mean ± SD) **	1.937 ± 0.622	1.980 ± 0.311	0.923
**Uterine Artery Resistance Index (RI) **	**Left (Mean ± SD) **	0.766 ± 0.139	0.766 ± 0.139	0.474
	**Right (Mean ± SD) **	0.786 ± 0.108	0.712 ± 0.089	0.206

**Table:** Table2. Predictors of Successful MTX Treatment in EP.

**Predictor**		Odds Ratio	Std. Err.	p-value	95% Confidence Interval
**Patient Age**		0.808	0.106	0.104	0.626 - 1.044
**Gestational Age**		1.006	0.0488	0.902	0.915 - 1.106
**History of EP**		0.529	0.641	0.599	0.049 - 5.672
**Observation of Tubal Ring**		6.500	6.953	0.080	0.799 - 52.897
**Hematoma Presence**		1.143	1.181	0.897	0.151 - 8.654
**Endometrial Thickness**		1.317	0.205	0.077	0.971 - 1.786
**Uterine Artery Pulsatility Index (PI)**	**Right**	1.174	1.089	0.863	0.191 - 7.227
	**Left**	1.966	1.344	0.322	0.515 - 7.505
**β-hCG Level**		1.000	0.0004	0.654	0.999 - 1.001
**Observation of Cul-de-Sac Fluid**		0.436	0.516	0.483	0.043 - 4.436

The results of this study may have limited applicability to broader populations due to the specific characteristics of the study cohort. A total of 100 patients were included, with 60 achieving successful treatment with a single dose of methotrexate, resulting in a success rate of 93.75% for ectopic pregnancy (EP) management. In the successful treatment group, the mean age was 31.84 ± 4.59 years, while the failed treatment group had a mean age of 34.50 ± 1.71 years, with no significant difference observed (P = 0.272). The mean parity in the successful group was 1.45 ± 1.54 compared to 2.25 ± 1.50 in the failed group, also showing no significant difference (P = 0.203). Similarly, the mean gravidity was 3.25 ± 1.50 in the successful group versus 2.50 ± 1.29 in the failed group, with no significant difference (P = 0.259). The mean gestational age in the successful treatment group was 37.00 ± 6.63 days, compared to 39.26 ± 12.80 days in the failed treatment group, with no significant difference (P = 0.732). The mean β-hCG level was 1395 ± 1464 IU/L in the successful group and 1135 ± 481.5 IU/L in the failed group, with no significant difference (P = 0.806). The frequency of IUD usage was 8% in the successful group and 0% in the failed group, with no significant difference (P = 0.547). The frequency of prior EP was 15% in the successful group and 0% in the failed group, also showing no significant difference (P = 0.900). The presence of hematoma was observed in 53% of the successful group and 50% of the failed group, with no significant difference (P = 0.900). The frequency of tubal ring observation was 86% in the successful group and 50% in the failed group, with no significant difference (P = 0.111). Additional details of other variables studied are provided in Table-1. To assess the relationship between key clinical features—such as history of EP, tubal ring presence, hematoma, and endometrial thickness—and treatment success, we conducted logistic regression analysis. As shown in Table-2, gestational age was not a significant predictor of successful treatment (odds ratio [OR]: 1.006, 95% confidence interval [CI]: 0.915 - 1.106, P = 0.902). A history of ectopic pregnancy demonstrated a non-significant association with treatment success (OR: 0.529, 95% CI: 0.049 - 5.672, P = 0.599). Although the reduced odds ratio suggests a potential trend, the wide confidence interval indicates high variability in the data. The presence of a tubal ring was borderline significant (OR: 6.500, 95% CI: 0.799 - 52.897, P = 0.080), suggesting that patients with tubal ring findings were approximately six times more likely to experience successful medical management compared to those without. The presence of hematoma was not a significant predictor of success (OR: 1.143, 95% CI: 0.151 - 8.654, P = 0.897). Endometrial thickness emerged as a borderline significant factor, with an odds ratio of 1.317 (95% CI: 0.971 - 1.786, P = 0.077).

Right pelvic inflammatory disease (PID) was not a significant predictor (OR: 1.174, 95% CI: 0.191 - 7.227, P = 0.863). Similarly, left PID showed no significant association with successful treatment (OR: 1.966, 95% CI: 0.515 - 7.505, P = 0.322). HCG levels did not significantly predict treatment success (odds ratio [OR]: 1.000, 95% confidence interval [CI]: 0.999 - 1.001, P = 0.654). Similarly, the presence of fluid in the cul-de-sac did not demonstrate a significant effect on treatment success (OR: 0.436, 95% CI: 0.043 - 4.436, P = 0.483). Patient age exhibited a negative but non-significant association with treatment success (OR: 0.808, 95% CI: 0.626 - 1.044, P = 0.104). Although older patients may have slightly lower odds of successful treatment, this finding was not statistically conclusive. The observed negative association between patient age and treatment success, while not statistically significant, raises important considerations for clinical practice. Older patients may face marginally lower odds of successful treatment, potentially due to age-related factors such as decreased ovarian reserve or altered pharmacokinetics of methotrexate. Clinicians should be mindful of these potential differences and consider them when counseling older patients about treatment options and expectations.

## Discussion

The results of the present study indicate that single-dose methotrexate (MTX) therapy for the treatment of tubal ectopic pregnancy (EP) achieves a high success rate. However, given the limited sample size, none of the examined variables demonstrated a significant impact or predictive power on treatment success. This finding suggests that while MTX therapy is effective, the small sample may restrict our ability to draw definitive conclusions regarding the influence of specific clinical factors on treatment outcomes. Future studies with larger sample sizes are needed to better understand the predictors of success in this context.

MTX (Methotrexate) is a folic acid antagonist that inhibits the synthesis of new cellular DNA. This antineoplastic and antimetabolic drug has been increasingly used for the treatment of EP since it was first reported by Tanaka and colleagues in 1982 [13]. For many patients, single-dose systemic MTX protocols are commonly employed as a standard treatment option, with no significant difference in success rates. Various studies in different populations have reported the success rate of single-dose methotrexate in treating EP to be as high as 89%, as noted in the study by Bottin et al. [14]. In the present study, the success rate of single-dose MTX treatment was reported to be 93.75%, which falls within an acceptable range and is higher and more effective compared to other studies. Proper patient selection for medical treatment and early detection of EP are likely factors that ultimately result in a positive impact on the success rate of medical treatment. This is because the longer the time since the onset of symptoms, the higher the likelihood of requiring surgical intervention.

In the present study, maternal age, parity, and gravidity parameters showed no significant association with the effectiveness of single-dose MTX therapy. This finding aligns with the results of other studies, such as those by Ghanaie et al. [15] and Mirbolouk et al. [16], where these parameters also lacked predictive power. No opposing studies have been reported in this regard, and various sources have not reported a significant effect of these parameters on the efficacy of MTX treatment for EP. The presence of a hematoma in ectopic pregnancy (EP) can negatively impact the success of methotrexate (MTX) treatment and increase the likelihood of requiring surgical interventions [17]. In some studies, such as that by Chegini et al. [17], the presence of a hematoma has demonstrated predictive power. However, in most studies, these parameters did not show predictive value for the success of medical treatment with MTX [18][5]. Similarly, in the present study, no significant association between hematoma presence and treatment success was reported, aligning with the majority of studies.

In the present study, the size of the EP was larger in the unsuccessful treatment group compared to the successful treatment group, but this difference was not statistically significant. These findings are consistent with studies by Arafa et al. [18] and Mirbolouk et al. [16], where the EP size was also larger in the unsuccessful treatment groups. This aligns with the understanding that larger ectopic masses are more resistant to medication, complicating treatment and increasing the need for further interventions.

In our study, the ampulla of the fallopian tube was the most frequent location of EP. However, the frequency of this variable did not significantly differ between the two study groups, which is consistent with other studies and reports indicating that the ampulla is the most common site of EP in women. Gestational age in our study did not have predictive value for the success of EP treatment with single-dose MTX. This finding is in line with studies by Bonin et al. [19], Mirbolouk et al. [16], and others, which also reported no significant differences in gestational age between successful and unsuccessful treatment groups.

In the present study, the serum β-hCG level at the start of treatment was 1395 in the responder group and 1135 in the non-responder group, with no significant difference between the two. Although the range of β-hCG levels in our study participants aligns with other studies, such as those by Shatkin Hamish et al. [20] and Ghanaie et al. [15], the lower levels in the unsuccessful treatment group cannot be explained except by the small sample size. This is because various studies have reported that serum β-hCG levels have predictive power for the success of EP treatment with single-dose MTX. In 2023, Ghanaie et al. reported that serum β-hCG levels at the start of treatment and their reduction on days 4 and 7 post-treatment can predict the success of single-dose MTX treatment [15]. Similarly, a 2024 study by Deniz et al. highlighted that the predictive power of β-hCG levels, as well as their reduction by day 4, can guide decisions regarding the need for additional drug dose [21].

One of the other notable trends observed was the borderline significance of endometrial thickness and tubal ring findings. An increased endometrial thickness was associated with higher odds of successful treatment, which aligns with prior studies suggesting that endometrial thickness may play a role in the body’s responsiveness to medical management [22]. Similarly, the presence of a tubal ring was borderline significant, indicating its potential as a prognostic marker. However, these findings must be interpreted cautiously due to the p-values being slightly above the threshold for significance.

Interestingly, patient age also showed a non-significant trend toward a negative association with successful treatment. While older age has been linked to reduced reproductive outcomes in general, its specific role in predicting the success of medical management for ectopic pregnancies remains unclear.

The use of methotrexate as a medical approach for treating ectopic pregnancy has shown promising results. However, only a limited number of factors have been identified as predictors of its success, indicating the need for further research in this area.

### Study limitations

This study has several limitations that should be considered when interpreting the results:

1. Small Sample Size: The total number of participants (100 patients) is relatively small, particularly in the control group, which consisted of only 40 patients. This limited sample size may restrict the statistical power of the analyses and the ability to detect significant associations between clinical variables and treatment success.

2. Case-to-Control Ratio: The disproportionate case-to-control ratio (60:40) may affect the generalizability of the findings. An optimal case-to-control ratio is essential for robust conclusions in case-control studies, and the current ratio may limit the reliability of the comparisons made.

3. Single-Center Study: The study was conducted at a single institution, which may limit the diversity of the patient population. Results may not be generalizable to other settings or populations with different demographic or clinical characteristics.

4. Retrospective Data Collection: Data were extracted from archived patient records, which may introduce biases related to incomplete or inconsistent documentation. Retrospective studies are inherently limited by the quality of the available data.

5. Potential Confounding Variables: While we attempted to control for various clinical factors, there may be unmeasured confounders that could influence treatment outcomes. Factors such as patient adherence to follow-up, variations in treatment protocols, and individual responses to methotrexate were not fully accounted for.

6. Short Follow-Up Period: The follow-up period for assessing treatment success may not have been long enough to capture all relevant outcomes. Longer follow-up may be necessary to evaluate the long-term effectiveness and potential complications associated with MTX therapy.

7. Limited Predictive Power: Despite the high success rate observed, none of the examined variables demonstrated significant predictive power for treatment success, which may be attributed to the small sample size and the inherent variability in patient responses.

In conclusion, while the study provides valuable insights into the effectiveness of single-dose MTX therapy for tubal ectopic pregnancy, these limitations highlight the need for further research with larger, multi-center cohorts to validate the findings and explore the predictors of treatment success more comprehensively.

## Conclusion

Although the variables examined in this study did not demonstrate predictive power for EP treatment outcomes with MTX, various studies suggest that the success of methotrexate treatment for ectopic pregnancy is influenced by multiple factors. These include β-hCG levels, the presence of fetal cardiac activity, the number of doses required, patient clinical characteristics, and the timing of diagnosis. Identifying these factors can assist physicians in selecting more suitable patients for treatment and achieving better outcomes. The results suggest that clinicians can confidently recommend methotrexate to eligible patients, particularly those with lower initial hCG levels and without significant complications, as it may lead to favorable outcomes while minimizing the risks associated with surgery. Further research is essential to thoroughly investigate these factors and their impacts on treatment outcomes, with the goal of developing more effective treatment protocols. Moreover, future studies should focus on larger, multicenter trials to validate the results and improve the generalizability of the findings across diverse populations, confirming the effectiveness of methotrexate treatment and identifying any variations in outcomes based on demographic or clinical factors.

## Conflict of Interest

None.
